# Stated preferences of adolescents and young adults for sexual and reproductive health services in Africa: a systematic review

**DOI:** 10.1080/26410397.2025.2520682

**Published:** 2025-07-02

**Authors:** Melaku Birhanu Alemu, Richard Norman, Jaya Dantas, Daniel Gashaneh Belay, Tsegaye G. Haile, Gavin Pereira, Gizachew A. Tessema

**Affiliations:** a PhD Candidate, Faculty of Health Sciences, Curtin School of Population Health, Curtin University, Bentley, Australia; Lecturer, Department of Health Systems and Policy, Institute of Public Health, University of Gondar, Gondar, Ethiopia. *Correspondence*: m.alemu@postgrad.curtin.edu.au; b Professor, Faculty of Health Sciences, Curtin School of Population Health, Curtin University, Bentley, Australia; c Professor, Faculty of Health Sciences, Curtin School of Population Health, Curtin University, Bentley, Australia; d PhD Candidate, Faculty of Health Sciences, Curtin School of Population Health, Curtin University, Bentley, Australia; e PhD Candidate, Faculty of Health Sciences, Curtin School of Population Health, Curtin University, Bentley, Australia; f Professor, Faculty of Health Sciences, Curtin School of Population Health, Curtin University, Bentley, Australia; Professor, enAble Institute, Curtin University, Bentley, WA, Australia; g Associate Professor, Faculty of Health Sciences, Curtin School of Population Health, Curtin University, Bentley, Australia; Associate Professor, enAble Institute, Curtin University, Bentley, WA, Australia

**Keywords:** sexual and reproductive health, SRH, stated preferences, preferences, discrete choice experiments, adolescents and young adults, young people, youth, Africa

## Abstract

Adolescents and young adults (AYAs) constitute approximately 30% of the African population and face significant challenges in accessing sexual and reproductive health (SRH) services. Low service uptake, despite availability, may indicate service provision misalignment with AYAs' preferences. This reflects the health sector gap and will partly compromise AYAs' rights. This study synthesised stated preference studies on SRH services among AYAs in Africa, following the PRISMA 2020 guidelines. Searches were conducted across six databases (MEDLINE, EMBASE, PsycINFO, CINAHL, Scopus and Global Health) and Google Scholar for grey literature on 24 April 2024. The attributes used to measure SRH preferences were classified based on the Donabedian quality of healthcare framework. A risk of bias assessment was conducted to evaluate the quality of included studies. The review was registered in PROSPERO (CRD42023386944). From 8,329 identified records, 16 studies with 8,005 participants from six countries were included in the final analysis. The attributes used were related to the structural (44.3%), process (41.7%) and outcome-related (13.9%) dimensions. The most important attributes were the cost of services, effectiveness of treatment and treatment frequency. Conversely, the least important attributes were treatment side effects, treatment and medical test sample collection characteristics, provider characteristics (age, gender and profession), and incentive type and recipient. In conclusion, AYAs’ preferences were mainly influenced by cost, treatment effectiveness and incentive distribution methods. Policymakers need to develop affordable and effective SRH programmes with tailored incentives to align with AYAs’ preferences to improve service uptake. However, these insights reflect data from a limited range of African countries.

## Plain language summary

Adolescents and young adults (AYAs) account for around 30% of Africa's population. Yet, a substantial proportion of them face challenges in getting sexual and reproductive health (SRH) services. Although SRH services are part of primary healthcare services, many AYAs do not benefit from these services. A key reason for the low uptake of SRH services may be a mismatch between existing service arrangements and AYAs’ preferences. We reviewed available studies that were undertaken using hypothetical choice-based scenarios. We searched six bibliographic databases for published studies: MEDLINE, EMBASE, PsycINFO, CINAHL, Scopus and Global Health. We also searched Google Scholar for grey literature. We found 16 studies involving 8005 participants from six African countries. The review found that the cost of services, treatment types and visit frequency are key factors shaping AYA preferences. In contrast, we found that AYAs cared least about treatment side effects, the provider's profile, including age and gender, and incentives. In conclusion, AYAs perceived that they would choose SRH services based on cost, effectiveness and incentives. Policymakers should provide effective, affordable treatment options for AYAs to improve the utilisation rate of SRH services among AYAs in Africa. It is also worthwhile to provide incentives to attract AYAs to use SRH facilities in Africa.

## Résumé

Les adolescents et les jeunes adultes forment près de 30% de la population africaine et rencontrent d’importants obstacles pour accéder aux services de santé sexuelle et reproductive (SSR). Le faible recours aux services, en dépit de leur disponibilité, peut indiquer une inadéquation de la prestation des services avec les préférences des adolescents et des jeunes adultes. Cela reflète les lacunes du secteur de la santé et compromet en partie les droits de ce groupe de population. Cet examen a synthétisé les études sur les préférences déclarées en matière de services de SSR chez les adolescents et les jeunes adultes en Afrique, conformément aux directives PRISMA 2020. Des recherches ont été menées dans six bases de données (MEDLINE, EMBASE, PsycINFO, CINAHL, Scopus, Global Health) et Google Scholar pour la littérature « grise » le 24 avril 2024. Les attributs utilisés pour mesurer les préférences en matière de SSR ont été classés selon le cadre de Donabedian pour la qualité des soins de santé. Une évaluation du risque de biais a été réalisée pour estimer la qualité des études incluses. L’examen a été enregistré dans PROSPERO (CRD42023386944). Sur 8329 dossiers identifiés, 16 études portant sur 8005 participants de six pays ont été incluses dans l’analyse finale. Les attributs utilisés étaient liés aux dimensions structurelles (44,3%), procédurales (41,7%) et relatives aux résultats (13,9%). Les attributs les plus importants étaient le coût des services, l’efficacité du traitement et la fréquence du traitement. À l’inverse, les attributs les moins importants étaient les effets secondaires du traitement, les caractéristiques du traitement et du prélèvement des échantillons de tests médicaux, les caractéristiques des prestataires (âge, genre, profession), ainsi que le type d’incitation et le bénéficiaire de ces mesures. En conclusion, les préférences des adolescents et des jeunes adultes étaient principalement influencées par le coût, l’efficacité du traitement et les méthodes de distribution des incitations. Les décideurs politiques doivent mettre au point des programmes de SSR abordables et efficaces, avec des incitations adaptées pour s’aligner sur les préférences des adolescents et des jeunes adultes, de façon à améliorer l’utilisation des services. Néanmoins, ces informations reflètent des données provenant d’un nombre limité de pays africains.

## Resumen

Los adolescentes y jóvenes adultos (AJA) constituyen aproximadamente el 30% de la población africana y enfrentan considerables retos para acceder a los servicios de salud sexual y reproductiva (SSR). La baja aceptación de servicios, a pesar de su disponibilidad, podría indicar la desalineación de la prestación de servicios con las preferencias de AJA. Esto refleja la laguna en el sector salud y comprometerá parcialmente los derechos de AJA. Este estudio sintetizó los estudios de las susodichas preferencias con relación a los servicios de SSR entre AJA en África, después de publicadas las directrices PRISMA de 2020. El 24 de abril de 2024, se realizaron búsquedas en seis bases de datos (MEDLINE, EMBASE, PsycINFO, CINAHL, Scopus, Global Health) y en Google Scholar para la literatura gris. Los atributos utilizados para medir las preferencias de SSR fueron clasificados según el marco Donabedian de calidad de la atención a la salud. Se realizó una evaluación del riesgo de sesgo para evaluar la calidad de los estudios incluidos. La revisión se registró en PROSPERO (CRD42023386944). De los 8329 registros identificados, se incluyeron en el análisis final 16 estudios con 8005 participantes provenientes de seis países. Los atributos utilizados estaban relacionados con las dimensiones estructurales (44.3%), procesales (41.7%) y relacionadas con los resultados (13.9%). Los atributos más importantes eran: el costo de los servicios, la eficacia del tratamiento y la frecuencia del tratamiento. En cambio, los atributos menos importantes fueron: los efectos secundarios del tratamiento, las características del tratamiento y de la recogida de muestras de exámenes médicos, las características de los prestadores de servicios (edad, género, profesión), el tipo de incentivo y el beneficiario. En conclusión, las preferencias de AJA eran influenciadas principalmente por costo, eficacia del tratamiento y métodos de distribución de incentivos. Los formuladores de políticas deberían crear programas de SSR asequibles y eficaces, con incentivos adaptados para alinearse con las preferencias de AJA, a fin de mejorar la aceptación de los servicios. Sin embargo, estas perspectivas reflejan los datos de una gama limitada de países africanos.

## Introduction

Sexual and reproductive health (SRH) refers to a condition of physical, mental and social well-being in relation to all aspects of the reproductive system.^1^ Access to SRH services is not merely a health issue but a fundamental human right, as recognised by international bodies such as the World Health Organisation (WHO) and the United Nations (UN).^2^ These rights include the right to access quality SRH services without discrimination, coercion or violence and the right to make autonomous decisions about one's body and reproductive life.^2^ Yet, adolescents and young adults (AYAs) often face substantial barriers to accessing SRH information and care, leading to higher risks of sexually transmitted infections (STIs), unintended pregnancies, unsafe abortions and unmet needs for contraception.^1^ SRH services include health education, contraception, safe abortion, STI treatment and care for sexual violence and reproductive cancers,^3–5^ with the aim of promoting community well-being.

Ensuring equitable access to SRH services is critical to enable individuals to achieve a responsible, secure and fulfilling sexual life^6,7^ and to uphold the human rights of all individuals to control their reproductive health, especially the reproductive rights of young women.^2^ The 1994 International Conference on Population and Development (ICPD) emphasised the need to recognise reproductive rights as a fundamental aspect of improving reproductive health. This includes ensuring informed choices regarding sexual and reproductive health (SRH) services, such as the number and spacing of children and the prevention of unintended pregnancies.^8,9^

Adolescence is a transitional period from childhood to adulthood. In this period, many behavioural and emotional changes are seen among young people.^10^ While the exact onset of adolescence remains debated, the UN and WHO define adolescents as individuals between the ages of 10 and 19 years,^11^ and young adults from 20 to 24 years.^12,13^ The physiological, psychological and social changes that occur during adolescence, including risky sexual activity, can increase the likelihood of developing sexual and reproductive health (SRH) issues.^14,15^

According to the United States Census Bureau estimate, around 480 million AYAs (aged 10–24 years) were living in Africa in 2025, which accounts for 31% of the African population.^16^ Although there have been some improvements in SRH services over the past decade, AYAs continue to be largely underserved in low-income countries.^17^ AYAs are often confronted with challenges regarding their sexual and reproductive health,^18^ with particular vulnerability to several SRH-related issues, including risky sexual behaviour, unintended pregnancy, unsafe abortions and STIs, including HIV/AIDS.^18,19^ For instance, despite the adverse perinatal and maternal outcomes of early pregnancy and childbearing that include preterm birth, low birth weight babies, maternal mortality and morbidity,^20–22^ approximately 12 million adolescent girls (aged 15–19) and 777,000 girls under 15 years give birth each year in low- and middle-income countries.^20,23,24^ In addition, in 2023, around 1.5 million adolescents (aged 10–19) were living with HIV worldwide, with approximately 140,000 new HIV infections.^25^ In the same year, about 1.9 million AYAs (aged 15–24) women lived with HIV globally, with around 4,000 new cases reported weekly, 78% of which occurred in sub-Saharan Africa.^26^

Preferences are demonstrated through the way individuals rank alternatives according to their relative importance. These preferences arise from the respondent's values, tastes and experiences, and are typically expressed through their selection of the most favourable option in hypothetical choice experiments.^27,28^ The low uptake of healthcare services among the AYA population is primarily due to the lack of availability of services as well as the inadequacy of the available services in addressing their unique needs.^19^ As the healthcare preferences of AYAs differ from those of older individuals,^29^ the involvement of AYAs (through measuring and taking into account their preferences) in the design of SRH services could lead to enhanced satisfaction, improved experiences, and positive economic and clinical outcomes.^30^ Considering the preferences of the public or patients in policy decisions can aid in better adoption and implementation of policies,^31,32^ leading to an efficient allocation of resources for providing SRH services. The provision of SRH services should also consider literacy and decision-making ability.^30^ Since the physiological and developmental changes in AYAs increase their tendency to engage in risky behaviours,^33^ including risky sexual behaviour,^34^ tailored services are essential to effectively translate policies into practice and advance the realisation of their sexual and reproductive rights.

Stated preference methods are used to understand how people value services by asking them hypothetical questions.^35^ Discrete choice experiments (DCEs) are among the most commonly used stated preference methods in healthcare.^36^ It is assumed that the utility an individual obtains from choosing one option over another is the cumulative utility derived from the underlying attributes of that option.^31^ In rational choice, respondents choose one option over the other to maximise their utility.^32^

Previous stated preference evidence has focused on specific SRH services in individual African countries.^37–41^ However, there is no summarised evidence on the preferences of AYAs for SRH services in Africa. Therefore, our study synthesised the available evidence of AYAs' preferences for SRH services in Africa, identifying the most and least important attributes that influence SRH service choice among AYAs in Africa. Understanding these preferences is essential for designing rights-based SRH services that respect, protect and fulfil the sexual and reproductive rights of AYAs.

## Methods

We followed the PRISMA (Preferred Reporting Items for Systematic Reviews and Meta-Analyses) 2020 reporting guideline.^42^ The review was guided by a prospectively registered protocol in PROSPERO (ID: CRD42023386944)^43^ and a review protocol.^44^

### Eligibility (inclusion and exclusion) criteria

We applied the population, intervention, comparison and outcome (PICO) approach to report the study selection criteria systematically. Studies that assessed AYAs' stated preferences for SRH services in Africa were included.

#### Population

Adolescents and young adults (AYAs) aged 10–24 years. Studies with overlapping age categories were excluded unless they provided a separate subgroup analysis specifically for AYAs. However, in some countries and contexts, young adults are considered individuals up to the age of 30 years, so we extended the age category accordingly in these cases.

#### Intervention

Hypothetical scenarios for sexual and reproductive health services (STIs including HIV/AIDS, family planning services, abortion care, cervical and breast cancer screening and treatment, sexual health education and counselling). Studies conducted to assess broad health status or well-being were excluded.

#### Outcome

The primary outcome was stated preferences for SRH services. The secondary outcomes included the relative importance of attributes, willingness to pay, uptake rate and preference heterogeneity. Studies that report a ranking of characteristics and opinions of respondents were excluded.

#### Type of studies

Primary stated preference studies using preference elicitation methodologies such as discrete choice experiments (DCEs), best-worst methods (BWM) and Thurston scaling were included. Published articles and grey literature, such as government reports and dissertations, were included. Study protocols, commentaries, conference abstracts and proceedings, and reviews were excluded from the analysis.

#### Context

Studies conducted in the area of SRH in one or more of the African countries were included. Studies that reported combined results of African and non-African countries were excluded unless they had a separate result for the African country.

#### Year and language

We included studies published in English after 2010 to ensure our findings reflect recent evidence on AYAs' preferences for SRH services in Africa. As preferences are shaped by changing experiences, societal shifts and health system developments, more recent studies were considered to capture current preferences.

### Information source

#### Database and search strategy

Articles were searched for in electronic databases including MEDLINE, EMBASE, PsycINFO, CINAHL, Scopus and Global Health through Ovid or EBSCO. In addition, we conducted a “snowball search” in Google Scholar using five pre-identified studies^37,38,41,45,46^ to identify grey literature.^47^ The first 100 results (10 pages of Google Scholar) were included for each study.

### Search strategy

The concepts for the search were organised into four main groups: (i) preference terms; (ii) youth, young adult and adolescent terms; (iii) sexual and reproductive health terms and (iv) terms relating to Africa. Keyword and subject heading searches were conducted using Boolean operators, truncation, wildcards and proximity operations (Supplementary file 1). The final search was conducted on 24 April 2024.

### Study selection

The search results from the included databases and Google Scholar were exported to EndNote to eliminate duplicates. Following this, the articles were transferred to Rayyan,^48^ a web-based screening tool, for initial screening based on their title and abstract. During this phase, two independent investigators, MBA and DGB, screened the articles. Disagreements were resolved through discussion and consultation with the third reviewer, GAT. All the studies that were selected from the title and abstract phase were exported to EndNote for a full-text review. MBA and DGB conducted the full-text screening independently, and the research team randomly verified 20% of the full-text screening process.

### Data extraction

The data extraction was performed by the MBA. The extracted data were reviewed by GAT and RN to ensure accuracy. In case of any disagreements, the team resolved them through discussion. We extracted data based on study characteristics, participant-related characteristics and stated preference-related characteristics. Study-related characteristics included publication year, country, healthcare service, sample size and sampling technique. Participant-related characteristics, such as age, gender, and educational status, were also collected. Additionally, we examined variables related to stated preferences, including methods of attribute development, the number and nature of attributes used, experimental design, data analysis model and the relative importance (most and least) of the identified attributes. The tool was developed by considering previous systematic reviews of discrete choice experiments^49,50^ and consulting with the investigators (MBA, GAT, RN, JD and GP). Data extraction was conducted using Excel (Supplementary file 2).

#### Study risk of bias assessment

The conjoint analysis checklists by ISPOR^31^ and PREFS checklists^51^ were used to assess the quality of the included studies. The ISPOR checklist had ten components (research question, attributes and levels, construction of choice tasks, experimental design, preference elicitation, instrument design, data collection, statistical analysis, results and conclusion, and study presentation).^31^ The PREFS checklist had five components, which included the purpose of the study, respondents' characteristics, explanations of the methods, findings and significance of the study.^51^ The quality assessment was conducted by two independent investigators, MBA and DGB (Supplementary file 3).

#### Data synthesis

The included studies were classified using the Donabedian model for healthcare quality (structure, process and outcome). The structure includes the physical and organisational aspects of healthcare settings, the process involves the methods of care delivery and outcome measures the effects on patient health.^49,52^ The most important and least important attributes were identified for the included studies. The most and least important attributes were identified by analysing the coefficient differences between the attribute levels. Attributes with the largest absolute differences were considered the most important, while those with the smallest absolute differences were deemed the least important.

### Ethics

Ethical approval was not required as the study is a secondary review of published and grey literature.

### Reflexivity statement

Our team comprises diverse experts in global health, sexual and reproductive health, and health economics. This diversity, reflecting a wide range of geographic experiences, genders, academic backgrounds and levels of seniority, significantly enriches our research, ensuring its relevance and impact. MBA, GAT, DGB and TH were born in Ethiopia and had extensive research experience in the African context, providing essential perspectives on the unique challenges of SRH services among AYAs. Their expertise ensures that our study remains grounded in the real-world experiences of AYAs in various African settings. MBA, DGB and TH have current academic affiliations at the university in Ethiopia. MBA, GAT, DGB, TH, GP and GAT are male researchers with extensive experience in maternal health and women's health. JD, a female senior academic, has extensive experience in gender research, sexual and reproductive health, and global health, and is from India and has lived and worked in Africa. While MBA, DGB and TH are junior researchers currently undertaking doctoral research, RN, JD, RN and GAT are senior academics contributing expertise in sexual and reproductive health (GAT, JD), global and community health and gender (JD), adolescent health (GAT, JD), health economics (RN) and biostatistics (GP) with extensive experience in supervision and multidisciplinary research. We have engaged in regular meetings throughout our research process to discuss research framing, analysis and findings. These diverse perspectives within our team produce methodologically rigorous, culturally sensitive findings and recommendations responsive to the needs of AYAs.

## Results

### Search results

**Figure 1.**
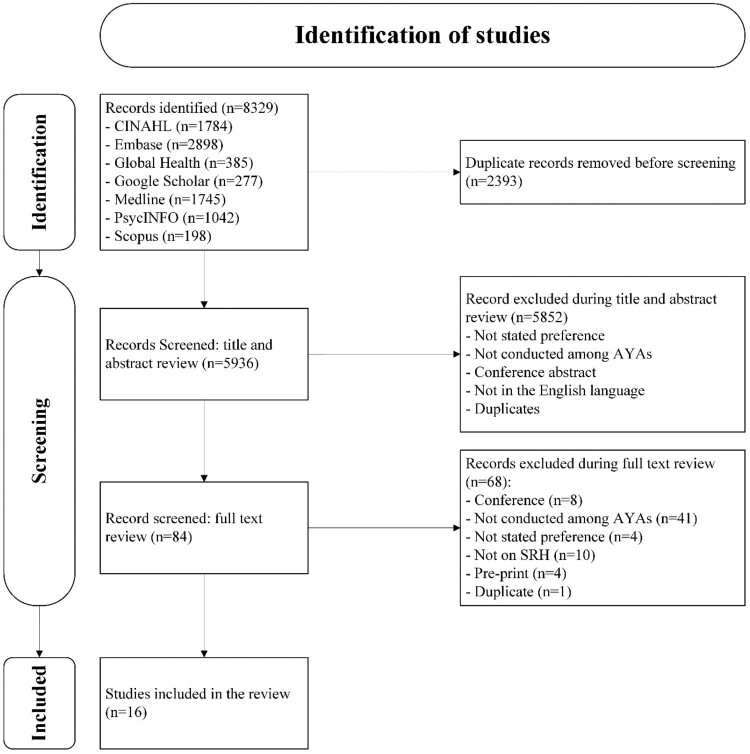
Preferred reporting items for systematic reviews and meta-analyses (PRISMA) flow diagram

A total of 8329 abstracts were identified from six databases and Google Scholar. After removing duplicates and ineligible articles during the title and abstract screening, 84 studies were eligible for full-text review. Sixteen studies met the criteria for inclusion in this analysis (Figure 1). Among these, four studies each reported a result from two countries. For the purpose of analysis, each country’s result was considered as an independent study.^40,41,53,54^. One study presented findings from three African countries (Eswatini, Kenya and South Africa) but did not disaggregate the results by individual country. Despite this limitation, the study was consistent across countries through study population recruitment, attributes and levels, a uniform discrete choice experiment (DCE) design, and the SRH service. Thus, the study was included as a single study in the review.^55^ A study conducted in Nigeria reported the results of two DCEs with different attributes and levels. As a result, it was considered two independent studies.^41^ Therefore, the analysis was conducted using a total of 20 studies.

### Study characteristics

All of the included studies used a discrete choice experiment to elicit the preferences of participants. Most included studies (*n *= 8) were conducted in South Africa^38,46^ (Table 1). A total of 8005 respondents participated in the included studies. The sample size ranges from 96 (Zimbabwe)^53^ to 807 (South Africa)^39^.

**Table 1.** Study characteristics of included studies (*n* = 20)

Study characteristics	Category	No	Percentage
Country	Kenya	2	10
	Malawi	2	10
	Nigeria	3	15
	South Africa	10	50
	Zimbabwe	2	10
	Multicountry*	1	5
Participants sex	Male and Female	12	60
	Female only	7	35
	Male only	1	5
Participants health condition	People living with HIV	2	10
	No health condition	18	90
Health services	HIV (testing, pre-exposure, counselling, treatment)	17	85
	Family planning	1	5
	SRH services (general)	2	10
Study population	Community	11	55
	Health facility	2	10
	School	2	10
	Mixed	5	5
Year of publication	2015–2017	4	20
	2018–2020	6	30
	2021–2024	10	50
*Multicountry: Eswatini, Kenya and South Africa			

### Risk of bias assessment

The quality of the included studies was assessed using the ISPOR conjoint analysis checklist and the PREFS checklist (see Supplementary File 3). Overall, most studies met the majority of ISPOR criteria, particularly in areas such as clearly defined research questions, appropriate attribute and level selection, and robust statistical analysis. However, several studies lacked adequate detail in explaining the experimental design and statistical analysis. Nearly half (n = 9) of the studies scored 90% or higher points in the ISPOR checklist. The PREFS assessment showed that while all studies clearly stated their purpose, respondent characteristics and significance of the study, some did not adequately describe relevant findings. Half of the studies (*n *= 11) fulfilled all the PREFS checklist items.

### Attributes and levels

Attributes are characteristics that are used to describe the SRH services. Each attribute has a level, and respondents were asked to choose between a bundle of different levels of attributes. All included studies utilised a literature review to identify the attributes and levels. Approximately 70% of the studies incorporated focus group discussions, while less than half (35%) employed expert consultations to identify the attributes and levels. A total of 115 attributes were used in the included papers. The median number of attributes per study was 5.5, ranging from five to eight attributes. Half of the studies utilised five attributes. Regarding attribute levels, three-quarters of the studies employed a minimum of two levels per attribute, ranging from two to four attribute levels. Approximately 65% of the studies used a maximum of four levels per attribute, ranging from four to eight attribute levels.

Structural, process and outcome-related attributes account for 44.7%, 38.8% and 16.5% of the total attributes. Almost all studies (*n *= 18) had at least one structural attribute. Likewise, all of the included studies had one or more process-related attributes, but only half of them (*n *= 7) had outcome-related attributes. (Table 2).

**Table 2.** Attribute classification of included papers based on the Donabedian quality framework

Author	Country	Attribute			Attribute number
		Structural	Process	Outcome	
Michaels-Igbokwe, Lagarde et al. 2015^44^	Malawi	4	2	0	6
Strauss, George et al. 2016^54^	South Africa	2	5	0	7
Indravudh, Sib et al. 2017^51^	Malawi	3	3	0	6
Indravudh, Sib et al. 2017^51^	Zimbabwe	4	3	0	7
Quaife, Eakle et al. 2018^55^	South Africa	1	1	4	6
Minnis, Browne et al. 2019^38^	Kenya	1	1	3	5
Minnis, Browne et al. 2019^38^	South Africa	1	1	3	5
Chetty-Makkan, Hoffmann et al. 2020^31^	South Africa	4	2	0	6
Galárraga, Kuo et al. 2020^56^	South Africa	4	1	0	5
Minnis, Atujuna et al. 2020^37^	South Africa	2	2	1	5
Montgomery, Browne et al. 2021^57^	South Africa	2	2	1	5
Ong, Nwaozuru et al. 2021^39^	Nigeria	3	2	0	5
Ong, Nwaozuru et al. 2021^39^	Nigeria	4	2	0	6
Eshun-Wilson, Akama et al. 2022^58^	Kenya	3	2	0	5
Little, Flomen et al. 2022^32^	South Africa	0	4	2	6
Browne, Manenzhe et al. 2023^52^	South Africa	2	3	0	5
Browne, Manenzhe et al. 2023^52^	Zimbabwe	2	3	0	5
Govathson, Long et al. 2023^59^	South Africa	4	4	0	8
Arije, Madan et al. 2024^60^	Nigeria	4	3	0	7
Little, Hanif et al. 2024^53^	Multicounty	0	3	2	5
**Total**		51 (44.3%)	48 (41.7%)	16 (13.9%)	115 (100%)
*Multicountry: Eswatini, Kenya and South Africa					

The structural dimensions include source of information, incentive (type and amount), location of services, health services availability and delivery mode, cost of services, profession of health provider, health provider characteristics (age, sex, residence) and health services accreditation (approved by WHO). The process-related attributes used include support provision, HIV testing and sample collection methods, the timing of incentive and treatment, opening hours, delivery location, frequency of use, confidentiality of services, protection duration and treatment characteristics (frequency of use, location of insertion, removability, duration of treatment). Finally, the outcome-related attributes include effectiveness and side effects of treatment, and disease prevention (Table 3).

**Table 3.** Attribute classification based on the Donabedian healthcare quality framework

Authors	Country	Attribute 1 and levels	Attribute 2 and levels	Attribute 3 and levels	Attribute 4 and levels	Attribute 5 and levels	Attribute 6 and levels	Attribute 7 and levels	Attribute 8 and levels
Michaels-Igbokwe, Lagarde et al. 2015^44^	Malawi	**Service provider gender (Male, Female)**	**Service provider age (Less than 30, >30)**	**HIV services availability (HCT only, HCT and ART)**	**Price (Free, 50MK, 150MK, 500MK)**	*Youth focus (Recreation and sports, music and drama, health education, no additional activity)*	*Confidentiality (Confidential, Sometimes not confidential)*	.	
Strauss, George et al. 2016^54^	South Africa	**Location (school, clinic, hospital, home)**	**Price (Free, 1.5USD, 3.75USD, 7.5USD)**	*How counselling is offered (individual counselling with nurse, group counselling with nurse, individual counselling with doctor, No counselling)*	*Methods used for testing (finger prick blood sample, intravenous blood draw, mouth swab, urine sample)*	*Timing of HCT (In the week after school, in the week during school time, Saturday, Sunday)*	*Duration (1hr, 30 min, 2 hrs, 3hrs)*	*Who conducted the HCT (nurse from community that you don't know, A nurse from community that you know, nurse from other community, doctor)*	
Indravudh, Sib et al. 2017^51^	Malawi	**Test price (Free, USD 0.07, USD 0.21)**	**Location (health facility, mobile clinic, home, home of provider)**	**Type of provider (Healthcare worker, lay distributor, intimate partner)**	*Sample collection (Oral-fluid, self tested blood-based, provider-delivered blood-based)*	*Pretest support (instruction leaflet, hotline, in-person, hotline and in-person)*	*Posttest support (Instruction leaflet, hotline, in person, hotline and in-person)*	.	
Indravudh, Sib et al. 2017^51^	Zimbabwe	**Test price (Free, USD 0.5, USD 1)**	**Provider age (<30 and equal,>30)**	**Provider residence (same community, outside of community)**	**Location (health facility, mobile clinic, home)**	*Pretest support (instruction leaflet, hotline, in-person)*	*Opening hours (regular hours, regular and evening and weekends)*	*Distribution (Batch distribution, individual distribution)*	
Quaife, Eakle et al. 2018^55^	South Africa	**Product (Oral pre-exposure prophylaxis, Microbicide gel, Microbicide gel with SILCS diaphragm, Vaginal ring, Injectable pre-exposure prophylaxis)**	*Frequency of use (every time you have sex, once per day, once per week, once per month, once per 3 months, once per 6 months, once per year)*	Pregnancy protection (yes, no)	HIV Protection (55%, 75%, 95%)	Protection for other STI (Yes, No)	Side effect – probability of occurrence (Stomach cramps, Nausea/feeling sick, Dizziness, None)		
Minnis, Browne et al. 2019 ^38^	Kenya	**Product form (ring, oral tablet, injection)**	*Frequency of use (every day, once per week, once per month, once per 2–3 month)*	Pregnancy prevention (No pregnancy prevention, pregnancy prevention and menstruation normal, pregnancy prevention and heavy and irregular menstruation, pregnancy prevention and may stop having monthly. Menstruation)	HIV prevention (90%, 70%, 50%, 30%)	Side effects (mild headache/dizziness, mild nausea/upset stomach, no side effects)	.		
Minnis, Browne et al. 2019^38^	South Africa	**Product form (ring, oral tablet, injection)**	*Frequency of use (every day, once per week, once per month, once per 2–3 month)*	HIV prevention (90%, 70%, 50%, 30%)	Pregnancy prevention (No pregnancy prevention, pregnancy prevention and menstruation normal, pregnancy prevention and heavy and irregular menstruation, pregnancy prevention and may stop having monthly. Menstruation)	Side effects (mild headache/dizziness, mild nausea/upset stomach, no side effects)	.	.	
Chetty-Makkan, Hoffmann et al. 2020^31^	South Africa	**Source of HIV information (social media, SMS text, Pamphlet)**	**Incentive amount (None, R25, R50)**	**Incentive type (Food voucher, Data bundle or airtime, cash)**	**Location of testing (Clinic, Home, school)**	*Support for HIV testing (alone, with friend, parents)*	*Testing method (Oral swab, finger prick)*	.	
Galárraga, Kuo et al. 2020^56^	South Africa	**Economic incentive (R0, R240, R480, R960, R1920)**	**Incentive format (n/a, cash, food voucher, fashion voucher)**	**Delivery mode (n/a, clinic, virtual/electronic delivery)**	**Economic incentive recipient (n/a, YLPWH only, YPLWH and parent/caregiver)**	*Participants (n/a, adherent & non-adherent youth, non adherent youth only)*	.	.	
Minnis, Atujuna et al. 2020^37^	South Africa	**Product form (injection, implant)**	**Site (clinic, pharmacy, community distribution, mobile clinic)**	*Dosing frequency (two, six, twelve months)*	*Delivery location (arm, buttock, thigh)*	Pain (mild, moderate)	.	.	
Montgomery, Browne et al. 2021^57^	South Africa	**Product form (One injection, two injections, implant)**	**Availability (pharmacy, health clinic, mobile clinic, community site like library or community hall)**	*Duration (One dose every 2 months, one dose every 6 months, one dose every year)*	*Location of injection/implant, (buttock, thigh, upper arm)*	Soreness after injection (mild and moderate)	.	.	
Ong, Nwaozuru et al. 2021^39^	Nigeria	**Out-of-pocket cost (Naira) (free, 500, 1000, 2000)**	**Location of the test (private hospital, public/government hospital, community health centre, NGO, sexual health clinic, awareness event, pharmacy or chemist, home or self-testing)**	**Availability of HIV Medicine (at location of testing, go to different location)**	*A person who tests (Doctor, Nurse, Trained healthcare volunteer, Yourself)*	*Type of test (blood test from arm (venipuncture), blood from finger prick, a swab from the mouth)*	.	.	
Ong, Nwaozuru et al. 2021^39^	Nigeria	**Out of pocket cost (Naira) (free, 500, 1000, 2000)**	**Test quality (Not approved by WHO, approved by WHO)**	**Location of the test (private hospital, public/government hospital, community health centre, NGO, sexual health clinic, awareness event, pharmacy or chemist, online order with home delivery)**	**Extra items (test for other STI (syphilis chlamydia and gonorrhoea), Pregnancy test-condoms and the contraceptive pill, information about safe sex;HIV and other STI, Malaria or tuberculosis test)**	*Support if tested positive (Flyer with a list of HIV clinics, online chat with a trained counsellor or youth health worker, same day in-person consultation with trained counsellor or youth health worker, same day in-person consultation with a doctor)*	*Type of test (blood from finger prick, swab from mouth)*	.	
Eshun-Wilson, Akama et al. 2022^58^	Kenya	**Value of gift (100, 300, 500 KSH)**	**Gift distribution (case, mobile money payment, airtime, shopping voucher)**	**Eligible for the gift (all youth, youth with time and virally suppressed)**	*Who collects the gift (only you, you and other elected person)*	*Timing of gift (each visit, end of the year)*	.	.	
Little, Flomen et al. 2022^32^	South Africa	*Protection duration (6, 12, 24 months)*	*Removability (Dissolves in body, dissolves in body and can be removed surgically within 1 month of insertion, Can only be surgically removed)*	*Number of insertions (one insertion, two insertions)*	*Location of insertion (buttocks, belly, thigh, arm)*	Side effects (minor pain at the insertion site, Pain at the insertion site and headache for days, Pain at the insertion site and headache for weeks)	Effectiveness for HIV (70%, 80%, 90%)	.	
Browne, Manenzhe et al. 2023^52^	South Africa	**Size (short, long)**	**Flexibility (Flexible, stiff)**	*How long it lasts (6 month, 1, 2 years)*	*Number of rods (1 rod, 2 rod)*	*Implant removal (Remove at clinic, No removal)*			
Browne, Manenzhe et al. 2023^52^	Zimbabwe	**Size (short, long)**	**Flexibility (Flexible, stiff)**	*How long it lasts (6 month, 1, 2 years)*	*Number of rods (1, 2 rod)*	*Implant removal (Remove at clinic, No removal)*			
Govathson, Long et al. 2023^59^	South Africa	**Location (school, clinic, community, private)**	**Health care provider (young within, young outside, old within, old outside)**	**Service (Condom only, contracetive & FP, HTC only, all health service)**	**Cost (free, R1–50, R51–100, >R100)**	*Confidentaility (Confidential, none)*	*Incentive (none, youth only, WIFI, food)*	*Time (morning, afternoon, Evening, weekends)*	*Staff attitude (friendly, unfreindly)*
Arije, Madan et al. 2024^60^	Nigeria	**Type of staff (Doctor, Nurse, Other health worker)**	**Physical enviroment (Very clean, moderately clean, Not clean at all)**	**Cost of service (Free, N500,N1500,N2500)**	**Contraceptive avilablity (at least one method always avilable, not always avilable)**	*Waiting time (No waiting, 30, 60 min)*	*Staff attitude (Open and friendly, stern and Jugmental)*	*Open hours (Weekdays only(8AM-4PM), Weekdays and after hours(8AM-8PM), Weekdays and weekends)*	
Little, Hanif et al. 2024^53^	Eswatini, Kenya, and South Africa	*Format (Injection with 2 months of protection, Injection with six months of protection, Surgically inserted implant with 12 months of protection that dissolves, Surgically inserted implant with 12 months of protection that has to be surgically removed)*	*Number of Insertions or injections for a full dose (One insertion/injection, Two insertions/injections)*	*Location of insertion (Arm, Belly, Buttocks)*	Feel (Palpable/can feel the product under the skin, Not palpable/cannot feel product under the skin)	Type of Protection (HIV only, HIV and contraception, HIV & STIs)			
**Note: Bold: Structure-related attributes;***Italic: Process-related attributes***and**Underlined: outcome-related attributes.									

### Construction of tasks and experimental design

**Figure 2.**
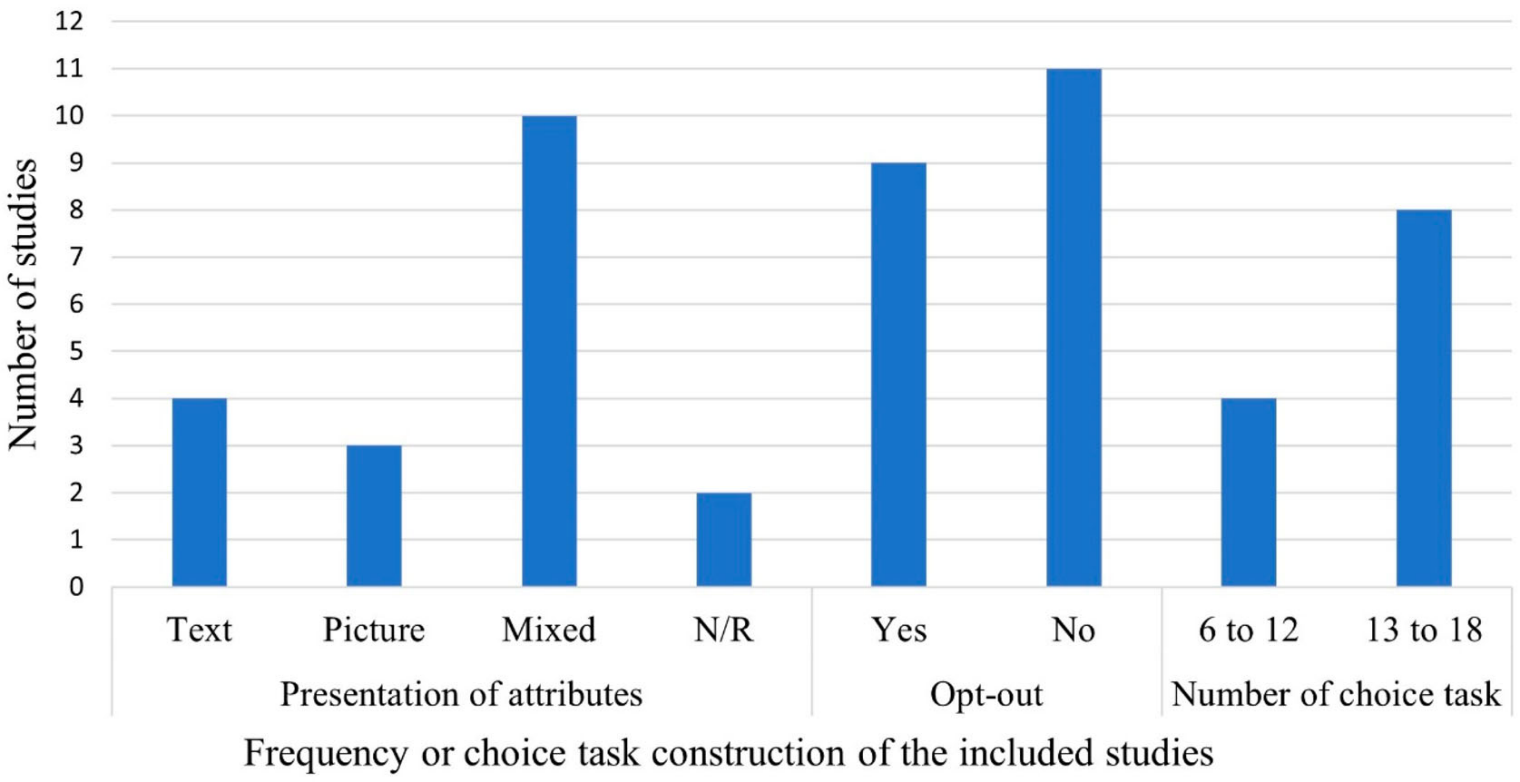
Construction of the discrete choice experiment questionnaire. N/R: Not reported

The levels of the attributes for the DCEs were presented in the form of text, pictures, emojis, or mixed forms. Half of the studies (*n *= 10) used a mixed form of attribute-level presentation. The median number of choice experiments each respondent answered was 9, ranging from 6 to 18. Regarding the choice option presented, half of the studies (*n *= 11) had the option of not choosing a service (opt-out), while the others required participants to pick one of the available options (i.e. forced design). Consequently, it was not possible to estimate the acceptability of forced-choice designs due to the absence of an opt-out option in these studies (Figure 2).

### Statistical analysis and relative importance of attributes

The majority of DCEs (*n *= 16) in the included studies used beta logit coefficients to estimate the preferences of participants, followed by odds ratios (*n *= 3). The willingness to pay and acceptability rate were estimated by half of the studies (*n *= 10). Similarly, three out of four studies presented the relative importance of attributes. Finally, all of the studies accounted for heterogeneity in preference (mixed logit, latent class, interaction or subgroup analysis). However, around 30% of the studies exclusively used mixed methods to account for these preferences. (Table 4).

**Table 4.** Statistical analysis used in the included studies

Study characteristics	Category	No	Percentage
Software used for analysis	STATA	12	60
	Nlogit	8	40
Preference estimate	Coefficients	16	80
	Odds Ratio	3	15
	Utility ratio	1	5
Willingness to pay	Yes	10	50
	No	10	50
Acceptability rate	Yes	10	50
	No	10	50
Relative importance	Yes	15	75
	No	5	25
Heterogeneity accounted	Mixed methods	6	30
	Subgroup analysis	6	30
	Interaction	4	20
	Combination of methods*	4	20
*The combination of the latent class model, the mixed model, a subgroup analysis and an interaction term.			

**Figure 3.**
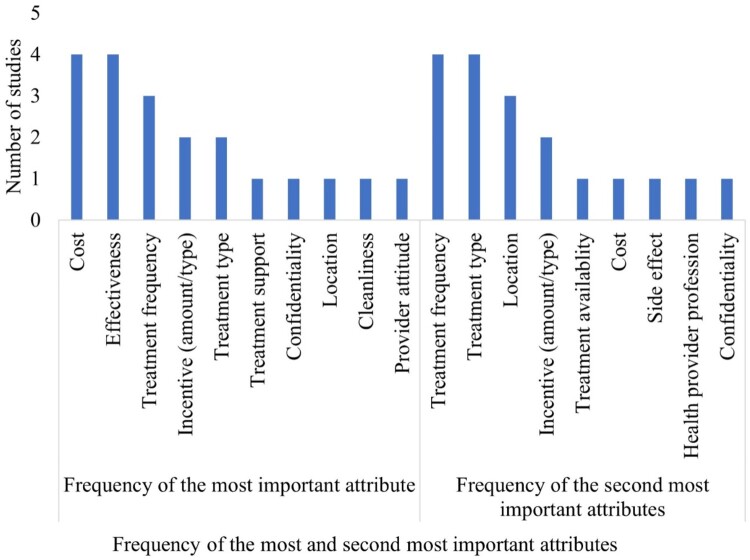
Most important attributes for SRH service use among adolescents and young adults in Africa

The majority of the most important attributes were related to structural and outcome-related factors. Price of service and incentives (*n *= 4) and effectiveness of treatment (*n *= 4) were the most important attributes for most of the papers. Incentives (amount and distribution methods), confidentiality, HIV testing support, treatment frequency and type, and location of SRH testing were the most important attributes identified in the remaining 12 studies. Furthermore, treatment availability (*n *= 4) and side effects (*n *= 4) were the second most important attributes in the included studies (Figure 3).

**Figure 4.**
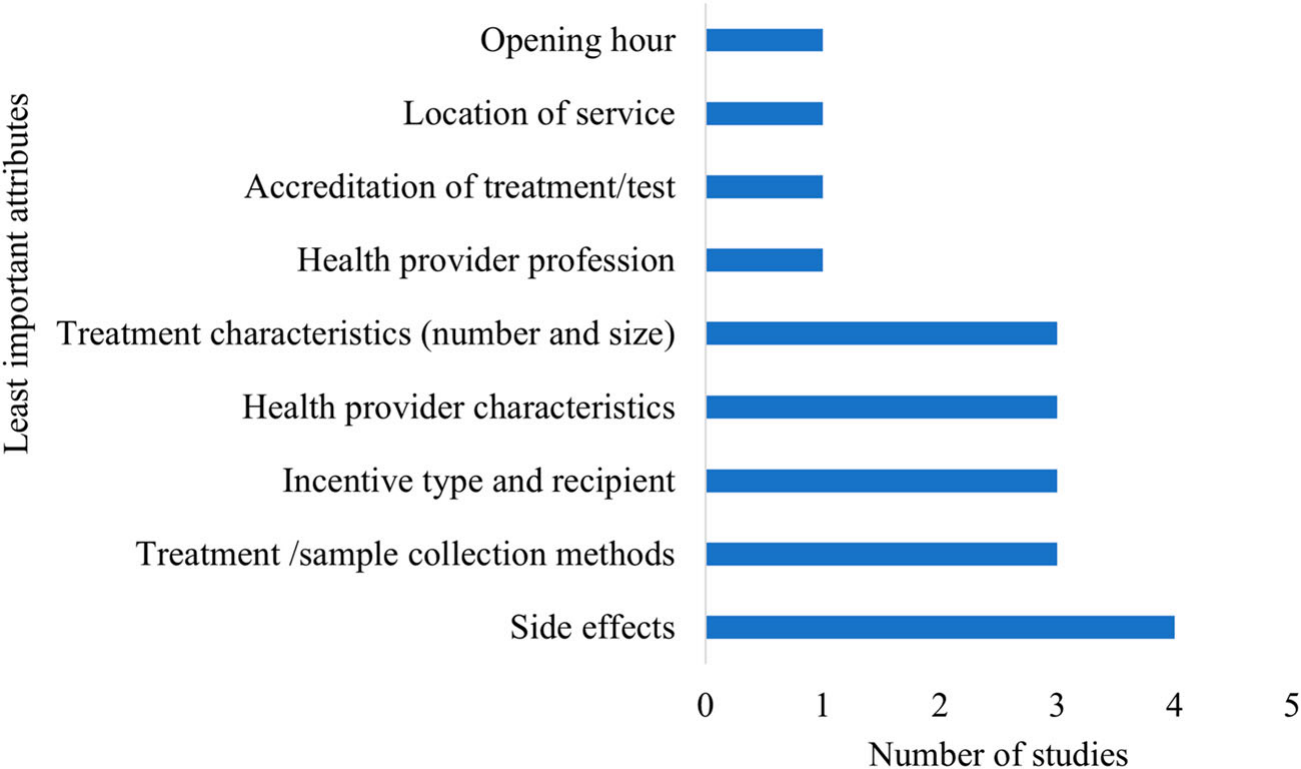
Frequency of the least important attributes for SRH service use among adolescents and young adults in Africa

Treatment side effects were found to be the least important attribute in four of the included studies. Attributes such as treatment /sample collection methods including the number of insertions for injection, and sample collection methods for HIV testing; incentive recipient (type and beneficiary); health provider characteristics (age and gender); and treatment characteristics (the number and size); were the least important attributes in three each of the included studies (Figure 4).

## Discussion

This systematic review of stated preference studies on SRH services among AYAs in Africa identified several attributes used to assess the preferences of AYAs for SRH services in Africa. The attributes were broadly categorised into (1) structural (e.g. incentives, service location, health services availability and services delivery mode, cost of services, profession and provider characteristics), (2) process (e.g. opening hours, and confidentiality), and (3) outcome-related attributes (e.g. treatment effectiveness). These are key aspects of health systems organisation that impact AYAs' decisions to utilise SRH services freely. Aligning services with their preferences is essential for improving service uptake and ensuring their right to accessible, acceptable and quality care, as outlined in international human rights frameworks.^56,57^

Most reported attributes were related to the structural aspects of the service provision. This was consistent with a stated preference research review in sub-Saharan Africa in maternal health services.^58^ The structural component is the major element of facility readiness, with health equipment and human resources.^59^ Different studies found attributes like service availability, cost of service and provider characteristics to affect adolescents' preference for SRH services.^60,61^ These findings have significant implications for health systems in Africa, where structural deficiencies often undermine service delivery.^62^ There is an urgent need to subsidise or fully provide SRH services free of charge to address cost barriers for AYAs. Furthermore, investments in strengthening health facility infrastructure and consistent supply chains for essential medicines are necessary to enhance the responsiveness of African health systems to the needs of young populations.

The process attributes were the second most used attribute classification. The process focuses on the care given to the client.^59^ A systematic review in sub-Saharan Africa stated waiting time and lack of confidentiality to be barriers to SRH utilisation.^63^ Factors such as treatment frequency, privacy, cleanliness and provider attitude were found to influence AYAs to utilise SRH services. Improving SRH service delivery in African health systems requires a shift towards more accessible and youth-centred models of care. For instance, reducing reliance on in-person appointments by incorporating online consultations and mobile health services can expand access while respecting AYAs’ right to confidential and convenient care. Ensuring privacy through dedicated consultation spaces and secure digital platforms is essential to safeguarding AYAs' confidentiality, an integral component of their SRH rights.^56,57^ Furthermore, training health providers to deliver compassionate, non-judgmental care is vital to fostering trust, upholding the right to respectful treatment, and increasing service utilisation among young people.

The outcome-related attributes, the effect of the health service on the health status, were the least used to assess the preference of AYAs for SRH.^59^ This was supported by a systematic review conducted in Sub-Saharan Africa.^58^ AYAs have mixed preferences for outcome-related attributes. Although treatment effectiveness was the most important attribute in four studies, side effects were the least important in others. Due to physiological changes, AYAs may be more willing than adults to take risks for benefits,^33^ which could explain their preference for highly effective services despite concerns about side effects. A review on contraceptive use noted that AYAs have varying preferences concerning the side effects of contraceptives.^64^ A study in Brazil found that the effectiveness of pre-exposure prophylaxis was more important than the side effects in influencing preference for HIV prophylaxis.^65^ Similarly, a review on HIV pre-exposure prophylaxis preferences highlighted perceived effectiveness as a key factor in the decision to use the service.^66^ In contrast to our review, a review of DCEs on cancer treatment, including reproductive cancers, stated that patients are more concerned about the side effects of treatment than the effectiveness of the treatment.^67^ The reason could be that cancer treatments could have devastating side effects compared to SRH treatments. Furthermore, the review predominantly involved adult populations from high-income countries, which may explain the variation in preferences, as adults in these settings may be more cautious about potential side effects, whereas AYAs in developing countries may prioritise other aspects of care. Therefore, offering personalised options could accommodate their varying preferences for side effects and other attributes. Additionally, emphasising the tangible benefits of SRH treatments can increase AYAs' engagement with SRH services, considering their tendency to take risks for perceived benefits.

Provider characteristic is the least important attribute that affects the choice of AYAs to utilise SRH services in Africa. This was consistent with a systematic review of family planning services among young people, which stated AYAs have a preference for provider attitude rather than provider characteristics like gender and age.^68^ SRH services in Africa should prioritise professionalism over demographic characteristics such as gender and age. Policies should focus on training healthcare providers to deliver non-judgmental, respectful, and youth-friendly care, ensuring that all AYAs receive services free from discrimination,^69^ regardless of their personal characteristics. This approach is essential to safeguarding AYAs' right to equitable healthcare.

Our review found that AYAs care about the incentive amount and type. A systematic review of HIV testing revealed that adolescent girls have the highest preference for HIV testing when a large number of incentives are offered, and they were less likely to take a test when the incentives are less.^70^ A study conducted in Mexico showed that incentive type and incentive amount were important attributes that affected the choice to take HIV prophylaxis.^71^ This implies that providing incentives, especially for underserved areas, could improve the utilisation of SRH services in Africa.

The risk of bias assessment highlights generally strong methodological quality across studies, supporting the reliability of the main findings. However, limited detail in experimental design and inconsistent reporting of results in some studies introduce potential bias. These issues may affect the transparency and generalisability of certain preference estimates. As such, while the overall conclusions remain robust, findings from less clearly reported studies should be interpreted with caution.

### Limitations of the study

All the studies originate from five countries: Malawi, South Africa, Zimbabwe, Kenya, and Nigeria. Consequently, the preferences of AYAs in Africa for SRH services may not be adequately represented by five countries. Additionally, one study was excluded from the final analysis because its results were embedded within the attributes,^45^ which made it difficult to extract the attribute levels. In addition, our review found that most of the studies on the preferences of AYAs in Africa were on HIV/AIDS and modern contraceptives. The results might not be representative of all SRH services, such as abortion. Furthermore, preferences can vary with age within the age range investigated in this study.

## Conclusion

The majority of the attributes used to measure the preferences of AYAs for SRH services were structure-related attributes. The price of the service, the effectiveness of the treatment, and the amount and distribution methods of incentives were found to be the most important attributes. In contrast, treatment side effects (independent of treatment effectiveness) were identified as the least important attribute. There is a paucity of studies from Africa, and those that were from Africa were limited to studies on HIV/AIDS. Policy makers should focus on availing effective SRH treatments with affordable prices and providing incentives for participation to meet the preferences of AYAs in Africa. Researchers are advised to broaden preference research to a wider range of SRH services for AYAs across various African countries.

### Author contributions

*Conceptualisation, MBA, RN, GP, JD and GAT. Data curation: MBA. Formal analysis: MBA. Investigation: MBA, DGB and TH. Methodology: MBA, RN, GP, JD and GAT. Project administration: RN, GP, JD and GAT. Resources: RN, GP, JD and GAT. Supervision: RN, GP, JD and GAT. Validation: MBA, DGB and TH. Visualisation: MBA. Writing – original draft: MBA. Writing – review & editing: RN, GP, JD, GAT, DGB and TH.*

*All authors approved the submission of the final manuscript*.

## Supplementary Material

Supplementary file 1. Search strategy.

Supplementary file 3. Quality appraisal.

Supplementary file 2. Data extraction tool

## Provenance statement

This article was not commissioned and went through external peer review.
