# Supplementary material for: Stated preferences of adolescents and young adults for sexual and reproductive health services in Africa: a systematic review
Source: Sex Reprod Health Matters. 2025 Jul 2;33(1):2520682. doi: 10.1080/26410397.2025.2520682 (PMC12320262; doi:10.1080/26410397.2025.2520682)
Supplement: Supplementary file 1. Search strategy. [file ZRHM_A_2520682_SM6050.docx]

Databases - PubMed/MEDLINE, EMBASE, PsycINFO, CINAHL, Scopus, Global Health and Google Scholar

Limits – English

|  | **CONCEPT 1 – Preference** | **CONCEPT 2 - SRH** | CONCEPT 3 - Youth | **CONCEPT 4 – Africa** |
| --- | --- | --- | --- | --- |
| **Keywords**  **OVID** | ((discrete or discrete-choice or stated or thurstone or conjoint or DCE or DCEs) ADJ3 (choice or experiment or valuation or method* or analysis or preference )).ti,ab. or  (preference* OR "stated-preference*" OR DCE or DCE* OR "best worst" OR BWM OR BWS OR conjoint* OR thurstone OR WTP OR "willingness to pay" OR "willingness to accept" OR WTA OR "patient weighting" OR "patient rating" OR "patient ranking" OR "patient perspective").ti,ab. | ((sexual OR reproductive OR maternal) ADJ3 (health* or problem* or function* or behavior* or behaviour* or care or education or information or service)) .ti,ab.  OR (SRH OR SRHR OR ASRH or intercourse  OR "sexually transmitted" OR STI* OR STD* OR HIV OR "human immunodeficiency virus" OR AIDS OR "acquired immunodeficiency syndrome" OR syphilis OR gonorrhoea OR gonorrhea OR chlamydia OR trachomatis OR trichomoniasis OR hepatitis OR HBV OR HCV OR herpes OR HSV OR papillomavirus OR HPV OR "genital wart*" OR antiretroviral*  OR "family planning" OR pregnan* OR contracepti* OR "birth control" OR LARC OR "intrauterine device*" OR IUD OR "intrauterine system" OR UPS OR sterili?ation OR vasectomy OR condom* OR OCP OR "morning after pill" OR diaphragm OR "unprotected sex"  OR abortion* OR "pregnancy loss*" OR miscarriage* OR "embryo loss" OR "pregnancy termination*"  OR childbirth or "child birth" or "maternal health" or prenatal or antenatal or postnatal  OR ((cervical or uterine or breast or testicular) ADJ3 (cancer* or neoplasm* or tumor* or tumour*))).ti,ab. | ((young or adolesc*) ADJ3 (people or person or adult or population or m?n or wom?n)).ti,ab.  OR (adolescent* OR teen* OR yout* OR minor* ) .ti,ab. | ("sub-saharan africa" OR "south of the sahara" OR "central africa*" OR "Western Sahara" OR Algeria or Angola or Benin or Botswana or "Burkina Faso" or Burundi or "Cabo Verde" or Cameroon or "Central African Republic" or Chad or Comoros or "Congo" or "Brazzaville" or "Kinshasa" or "Democratic Republic of Congo DRC" or "Cote d'Ivoire" or "Ivory Coast" or Djibouti or Egypt or "Equatorial Guinea" or Eritrea or "Eswatini" or "Swaziland" or Ethiopia or Gabon or Gambia or Ghana or Guinea or "Guinea-Bissau" or Kenya or Lesotho or Liberia or Libya or Madagascar or Malawi or Mali or Mauritania or Mauritius or Morocco or Mozambique or Namibia or Niger or Nigeria or Rwanda or "Sao Tome and Principe" or Senegal or Seychelles or "Sierra Leone" or Somalia or "South Africa" or "South Sudan" or Sudan or Tanzania or Togo or Tunisia or Uganda or Zambia or Zimbabwe).ti,ab. |
| **Keywords**  **EBSCO** | ((discrete or discrete-choice or stated or thurstone or conjoint or DCE or DCEs) N3 (choice or experiment or valuation or method* or analysis or preference )) or  (preference* OR "stated-preference*" OR DCE or DCE* OR "best worst" OR BWM OR BWS OR conjoint* OR thurstone OR WTP OR "willingness to pay" OR "willingness to accept" OR WTA OR "patient weighting" OR "patient rating" OR "patient ranking" OR "patient perspective") | ((sexual OR reproductive OR maternal) N3 (health* or problem* or function* or behavior* or behaviour* or care or education or information or service))  OR (SRH OR SRHR OR ASRH or intercourse  OR "sexually transmitted" OR STI* OR STD* OR HIV OR "human immunodeficiency virus" OR AIDS OR "acquired immunodeficiency syndrome" OR syphilis OR gonorrhoea OR gonorrhea OR chlamydia OR trachomatis OR trichomoniasis OR hepatitis OR HBV OR HCV OR herpes OR HSV OR papillomavirus OR HPV OR "genital wart*" OR antiretroviral*  OR "family planning" OR pregnan* OR contracepti* OR "birth control" OR LARC OR "intrauterine device*" OR IUD OR "intrauterine system" OR UPS OR sterili?ation OR vasectomy OR condom* OR OCP OR "morning after pill" OR diaphragm OR "unprotected sex"  OR abortion* OR "pregnancy loss*" OR miscarriage* OR "embryo loss" OR "pregnancy termination*"  OR childbirth or "child birth" or "maternal health" or prenatal or antenatal or postnatal  OR ((cervical or uterine or breast or testicular) N3 (cancer* or neoplasm* or tumor* or tumour*))) | ((young or adolesc*) N3 (people or person or adult or population or m?n or wom?n))  OR (adolescent* OR teen* OR yout* OR minor* ) | ("sub-saharan africa" OR "south of the sahara" OR "central africa*" OR "Western Sahara" OR Algeria or Angola or Benin or Botswana or "Burkina Faso" or Burundi or "Cabo Verde" or Cameroon or "Central African Republic" or Chad or Comoros or "Congo" or "Brazzaville" or "Kinshasa" or "Democratic Republic of Congo DRC" or "Cote d'Ivoire" or "Ivory Coast" or Djibouti or Egypt or "Equatorial Guinea" or Eritrea or "Eswatini" or "Swaziland" or Ethiopia or Gabon or Gambia or Ghana or Guinea or "Guinea-Bissau" or Kenya or Lesotho or Liberia or Libya or Madagascar or Malawi or Mali or Mauritania or Mauritius or Morocco or Mozambique or Namibia or Niger or Nigeria or Rwanda or "Sao Tome and Principe" or Senegal or Seychelles or "Sierra Leone" or Somalia or "South Africa" or "South Sudan" or Sudan or Tanzania or Togo or Tunisia or Uganda or Zambia or Zimbabwe) |
| Subject Headings | | | | |
| **Medline**  **(Mesh)** | exp Patient Preference/ or exp Choice Behavior/ or exp Decision Making/ or Patient Satisfaction/ | exp Sexually Transmitted Diseases/ or exp Sexual Health/ or Sex Education/ or Sexual Behavior/ or exp Reproductive Health Services/ or exp Reproductive Health/ or exp Abortion, Induced/ or reproductive behavior/ or exp Contraception/ or exp contraception behavior/ or exp Family Planning Services/ or Pregnancy Tests/ or exp HIV/ or Adolescent Behavior/ | exp Adolescent/ or exp Young Adult/ | exp "Africa South of the Sahara"/ or Comoros/ or Madagascar/ or Mauritius/ or Seychelles/ |
| **Embase** | exp patient preference/ or exp decision making/ or exp satisfaction/ | exp sexually transmitted disease/ or sexual education/ or sexual health/ or sexual behavior/ or exp sexuality/ or reproductive health/ or exp family planning/ or exp induced abortion/ or exp contraception/ or exp pregnancy test/ or exp Human immunodeficiency virus/ or exp adolescent sexual behavior/ or exp adolescent behavior/ or exp sexual behavior/ | exp juvenile/ or exp young adult/ or exp adolescent/ | exp "Africa south of the Sahara"/ or Comoros/ or Madagascar/ or Mauritius/ or Seychelles/ |
| PsycINFO | exp conjoint measurement/ or exp Client Attitudes/ or exp Consumer Behavior/ or exp Health Care Services/ or exp Health Behavior/ or exp Decision Making/ or exp Choice Behavior/ or exp Consumer Attitudes/ or exp Consumer Research/ or exp Choice Behavior/ or exp "Forced Choice (Testing Method)"/ | exp Sexually Transmitted Diseases/ or exp Sexual Risk Taking/ or exp HIV Testing/ or exp AIDS Prevention/ or exp Sex Education/ or exp Sexual Risk Taking/ or exp Sexual Health/ or exp Sexual Attitudes/ or exp Reproductive Health/ or exp sexual reproduction/ or exp Adolescent Pregnancy/ or exp Psychosexual Behavior/ or exp Induced Abortion/ or exp HIV/ | No term | No term |
| CINAHL | (MH "Patient Preference") or (MH "Forced Choice Scaling") or (MH "Decision Making+") or (MH "Health Behavior+") or (MH "Consumer Participation") | (MH "Sexually Transmitted Diseases+") or (MH "Sexual Health") or (MH "Health Services Needs and Demand") OR (MH "Student Health Services") or (MH "Reproductive Health") or  (MH "Contraception+") or (MH "Family Planning") OR (MH "Attitude to Sexuality+") OR (MH "Unsafe Sex") OR (MH "Attitude to Abortion") or (MH "Abortion, Induced+") or (MH "Pregnancy in Adolescence+") or (MH "Human Immunodeficiency Virus+") | (MH "Adolescence") or (MH "Young Adult") | (MH "Africa South of the Sahara+") or  (MH "Indian Ocean Islands+") |
| Scopus (Keywords) | ((discrete or discrete-choice or stated or thurstone or conjoint or DCE or DCEs) Pre/3 (choice or experiment or valuation or method* or analysis or preference )) or (preference* OR "stated-preference*" OR DCE or DCE* OR "best worst" OR BWM OR BWS OR conjoint* OR thurstone OR WTP OR "willingness to pay" OR "willingness to accept" OR WTA OR "patient weighting" OR "patient rating" OR "patient ranking" OR "patient perspective") | ((sexual OR reproductive OR maternal) Pre/3 (health* or problem* or function* or behavior* or behaviour* or care or education or information or service))  OR (SRH OR SRHR OR ASRH or intercourse  OR "sexually transmitted" OR STI* OR STD* OR HIV OR "human immunodeficiency virus" OR AIDS OR "acquired immunodeficiency syndrome" OR syphilis OR gonorrhoea OR gonorrhea OR chlamydia OR trachomatis OR trichomoniasis OR hepatitis OR HBV OR HCV OR herpes OR HSV OR papillomavirus OR HPV OR "genital wart*" OR antiretroviral*  OR "family planning" OR pregnan* OR contracepti* OR "birth control" OR LARC OR "intrauterine device*" OR IUD OR "intrauterine system" OR UPS OR sterili?ation OR vasectomy OR condom* OR OCP OR "morning after pill" OR diaphragm OR "unprotected sex"  OR abortion* OR "pregnancy loss*" OR miscarriage* OR "embryo loss" OR "pregnancy termination*"  OR childbirth or "child birth" or "maternal health" or prenatal or antenatal or postnatal)  OR ((cervical or uterine or breast or testicular) Pre/3 (cancer* or neoplasm* or tumor* or tumour*)) | ((young or adolesc*) Pre/3 (people or person or adult or population or m?n or wom?n))  OR (adolescent* OR teen* OR yout* OR minor* ) | ("sub-saharan africa" OR "south of the sahara" OR "central africa*" OR "Western Sahara" OR Algeria or Angola or Benin or Botswana or "Burkina Faso" or Burundi or "Cabo Verde" or Cameroon or "Central African Republic" or Chad or Comoros or "Congo" or "Brazzaville" or "Kinshasa" or "Democratic Republic of Congo DRC" or "Cote d'Ivoire" or "Ivory Coast" or Djibouti or Egypt or "Equatorial Guinea" or Eritrea or "Eswatini" or "Swaziland" or Ethiopia or Gabon or Gambia or Ghana or Guinea or "Guinea-Bissau" or Kenya or Lesotho or Liberia or Libya or Madagascar or Malawi or Mali or Mauritania or Mauritius or Morocco or Mozambique or Namibia or Niger or Nigeria or Rwanda or "Sao Tome and Principe" or Senegal or Seychelles or "Sierra Leone" or Somalia or "South Africa" or "South Sudan" or Sudan or Tanzania or Togo or Tunisia or Uganda or Zambia or Zimbabwe) |
| Global Health | exp decision making/ or exp consumer preferences/ or exp behaviour/ or exp willingness to pay/ or exp consumer attitudes/ or exp consumer behaviour/ | exp sexual health/ or exp reproductive health/ or human immunodeficiency viruses/ or sexual behaviour/ or HIV infections/ or acquired immune deficiency syndrome/ or sexually transmitted diseases/or Family planning/ or sexual health/ or sexual behaviour/ or health education/ or exp abortion/ or exp induced abortion/ or exp contraception/ | exp adolescents/ or exp young adults/ or exp youth/ | exp "Africa south of the Sahara"/ or Comoros/ or Madagascar/ or Mauritius/ or Seychelles/ |
| Google scholar | The first 100 articles from Google Scholar for the pre-identified five articles were searched.   1. Designing a package of sexual and reproductive health and HIV outreach services to meet the heterogeneous preferences of young people in Malawi: results from a discrete choice experiment 2. Designing HIV Testing and Self-Testing Services for Young People in Nigeria: A Discrete Choice Experiment 3. Youth Preferences for HIV Testing in South Africa: Findings from the Youth Action for Health (YA4H) Study Using a Discrete Choice Experiment 4. HIV Pre-exposure Prophylaxis Implant Stated Preferences and Priorities: Results of a Discrete Choice Experiment Among Women and Adolescent Girls in Gauteng Province, South Africa 5. Young People’s Preferences for Family Planning Service Providers in Rural Malawi: A Discrete Choice Experiment | | | |
