# Supplementary material for: Stated preferences of adolescents and young adults for sexual and reproductive health services in Africa: a systematic review
Source: Sex Reprod Health Matters. 2025 Jul 2;33(1):2520682. doi: 10.1080/26410397.2025.2520682 (PMC12320262; doi:10.1080/26410397.2025.2520682)
Supplement: Supplementary file 2. Data extraction tool [file ZRHM_A_2520682_SM6048.docx]

**Supplementary file 2: Data Extraction template**

| **Study context** | |
| --- | --- |
| General study characteristics (title, author(s): year published, citation) |  |
| Type of study (DCE, Best worst or Thurstone Scale) |  |
| Sources of funding |  |
| Competing interests |  |
| Setting (country, focus, disease condition) |  |
| Objective |  |
| Mode of survey administration |  |
| Ethical considerations |  |
| Inclusion/exclusion criteria |  |
| **Participant characteristics** | |
| Participants’ age (min-max, mean) |  |
| Participants sex |  |
| Participants’ pre-existing conditions (HIV, STI, CSW) |  |
| Education background |  |
| **Sampling** | |
| Sampling and recruitment |  |
| Sample population (patient, healthcare provider, general public) |  |
| Sample size |  |
| Were the sample size calculations carried out? |  |
| **Attributes and Levels of Development** | |
| Was a literature review conducted? |  |
| Consultation with experts in the field? |  |
| Focus group? If yes, what was the size of the focus group? |  |
| Interviews? If yes, how many interviews, and with whom? |  |
| Tested using a pilot study? If yes, how many participated? |  |
| Any other notable characteristics of attribute development |  |
| Number of attributes |  |
| List of attributes |  |
| List of levels for each attribute |  |
| Method of attribute presentation (picture, text only, emoji…) |  |
| **Survey design** | |
| Number of options presented per task |  |
| Number of attributes presented for each option |  |
| Was an opt-out option included? |  |
| Is a dominant choice question included? |  |
| Was there a block design? |  |
| Total number of questions - tasks (not including dominant choices) |  |
| Type of design (full factorial, partial factorial, etc.) |  |
| What software is used for experimental design? (Ngene, Sawtooth, SAS, etc.) |  |
| What criteria were used to judge the design? |  |
| Administration method (electronic, paper-based) |  |
| **Additional data collected** | |
| Demographic information collected? |  |
| Disease history collected? |  |
| Was any other data collected?  If so, what? |  |
| Anything else asked? If so, what? |  |
|  |  |
| **Analysis** | |
| What was the main method of analysis? |  |
| Any secondary method(s) of analysis? |  |
| Software used for analysis |  |
| Estimation procedure (probit, random effect probit, logit, random effect logit, CLM, mixed logit, latent scale, heteroskedastic …) |  |
| **Outcome measures** | |
| Were results presented using importance scores? |  |
| Were results presented using time until progression as numeraire? |  |
| Were results presented using money as numeraire (i.e. WTP)? |  |
| Were results presented using maximum acceptable risk? |  |
| Were results presented using a predicted probability of uptake? |  |
| Were results presented using utilities? |  |
| Was participant heterogeneity examined in any other way?  If so, what was examined? |  |
| **Conclusions** | |
| What was the most valued positive attribute? |  |
| What was the second most valued positive attribute? |  |
| What was the most valued negative attribute? |  |
| What was the second most valued negative attribute? |  |
| If looking at the mode of administration, what was the most preferred method? |  |
| If looking at the mode of administration, what was the least preferred method? |  |
